# Indents

**Published:** 1918-05

**Authors:** 


### INDENTS

**☞ Brief Articles of Technical or Scientific Value will receive notice and comment. Contributions invited.**

*Soldering* A minute quantity of gum arabic rubbed up with the borax and water on the slate will prevent the borax from frothing.—J. J. F. McManus, *Commonwealth Dental Review.*

*Expensive Expectoration* March 21, 235 persons were arrested, charged with violating the city ordinance prohibiting expectoration on sidewalks of Chicago and in public places. Of the accused 226 pleaded guilty, and were each fined $1.

*Investigate Powdered Glass Reports* The New York City Health Department has analyzed in its laboratory more than one hundred samples of bread, rolls, candy and other foodstuffs said to contain powdered glass. No glass was found. Fifty per cent. of the samples contained sand or grit. The department states that in normal times only five per cent. of persons finding sand or grit in their food would report it, while at the present time seventy-five or eighty per cent. would report the fact to the department. The department warns the public against the wave of hysteria that seems to be the result of these unfounded reports.—*Journal A. M. A.*

*Removing Guttapercha Root Fillings* Adjust the rubber dam carefully and remove filling from crown and pulp chamber with engine or otherwise. Then saturate a pledget of cotton with chloroform and place it over the orifice of the canal. Over this place some softened rubber or wax and force it as far as possible by pressure. Then work down the side of the filling with fine broaches, etc., removing all the guttapercha possible. Then wash the canal with chloroform on cotton wound on broaches until all traces of chloropercha disappear, being careful not to force anything through apical opening. The same technic can be used with proper variations for canals filled with other materials, as in the case of most canal paste sulphuric acid may be used in place of chloroform.—W. G. Ebersole, in *Dental Summary.*

*Dentin* I never expose freshly cut dentin to the fluids of the mouth when I can help it, and whether I do or do not I treat it to a touch of oil of cloves. Treating the cavity with alcohol followed by oil of cloves warmed with a puff of hot air from your chip blower, and the cavity stopped with guttapercha until the patient returns, will prevent the sensitiveness sometimes complained of after setting an inlay. Not only that, pulps are not so likely to die.—Homer Almon, in *Review.*

*Sterilizing the Apical Area* I have repeatedly heard the head of the dental research work in this country say that "nature has been very kind to us, gentlemen." I say to you that nature has not been "kind to us," but on the contrary she has been driven to make frantic efforts to protect herself against the outrages which we have been committing upon her, and now comes some of these men who add insult to injury when they advocate the necessity of sterilizing the tissue which lies just beyond the apex of the roots of nonvital teeth. To advocate such a line of procedure at this time is to advocate the committing of a crime against nature, for up to the present time *science has been unable to produce any method of destroying pathogenic micro-organic life in the tissue of the human body that is not*

*far more destructive and disastrous to the human tissue than it is to the microorganic life contained therein*—Dr. Ebersole, in *Summary.*

*To Sterilize Dentin* Before inserting a filling or cement base in a tooth in which the pulp is alive, evaporate a fifteen per cent. solution of thymol in alcohol in the cavity. The thymol will penetrate the tubuli and sterilize the dentin. This will avoid irritation to the pulp, which may cause secondary dentin. pulp nodules, or even death to the pulp.—A. de Vries, in *Review.*

*Diagnosis of Hemophilia* Dubois recommends that the lobe of the ear be pricked, and the drop of blood that will appear every thirty seconds is taken up with blotting paper. In a normal condition of the blood the hemorrhage will cease after ten to twelve blottings, owing to the formation of a thrombus.—*Cosmos.*

*Polishing Vulcanite Plates* After the plate has been filed, scraped and sandpapered, use a paste made of pumice, emery and medium fine silex in equal parts. By combining these grits in equal parts of beeswax and hard oil all dust will be avoided, and the abrasives are added as needed. Some care not to overheat the plate is necessary.—F. W. Frahm, in *Pacific Gazette.*

*Zinc Dies with Zinc Counters* I should like again to repeat my conviction of the efficiency of zinc counterdies. They can be quickly poured on to very hot zinc dies without any coating, provided that the molten zinc is just beginning to crystallize and no adhesion will take place. Having used no lead or tin counters for many years, I have no hesitation in again recommending this quick and efficient method to my colleagues.—H. J. Morris, *British Dental Journal.*

*Platinum Comman-deered* The government is planning to collect all platinum not needed in the essential industries to supply its needs in munition work. This will not affect reasonable requirements of dentistry, but means that all holdings not actually needed must be turned over to the government. Every dentist who has useless platinum in his possession should be glad to help the government out at this time.

*To Desensitize Teeth that Are Sensitive to Scaling* Dry sensitive part thoroughly. Apply for 4-5 minutes a hot saturated solution of potassium carbonate in glycerin on a pellet of cotton. Dry part with warm air. Repeat application leaving the cotton in place until the patient notices a burning sensation in the tooth, when the scaling may be continued painlessly. This solution will not cause discoloration.—A. de Vries, in *Dental Review.*

*Recovering Swallowed Dentures* Herbert Lilley describes in the *London Lancet,* February 23, an improved instrument for "endoscopy." Formerly the illumination was effected by a lamp from above, but Lilley employs the distal illumination, placing a lamp at the lower end of the tube. The patient lies on his back with the shoulders slightly raised and the head thrown back, thus bringing the mouth and pharynx nearly in a straight view. The patient may be or not anesthetized. The endoscope is adroitly introduced and the trachea or oesophagus brought directly into view. It is practicable to reach objects impacted even in the substance of the lungs, or in oesophagus by means of suitable forceps or other instruments.—*Dental Record.*

The house fly is a modest cuss,
He never seeks for fame,
He has no bus'ness in the soup,
But he gets there just the same.

*Dental Uses for Wounded Soldiers in England*

The Dental College in Liverpool, England, is cooperating with the Commissioner of Pensions in training discharged soldiers and sailors to become dental mechanics. Many soldiers who are otherwise incapacitated can render good service in this capacity, and earn satisfactory wages. There is need for this service and the government will to that extent be relieved of their support. The men would be happier because of a profitable employment and it would seem that this opening should make a strong appeal to such as are fitted for it.

*Efficiency of Dental Medicaments*

Dr. W. A. Price recently gave the results of his investigation of the value of the usually used root canal treatments to the Toronto Dental Society. Dr. Harold Clark is prompted to make the following comments:

"It has occurred to me that it might be wise to come back and look at the wreckage and see if there are not some pieces left that are worth carrying away.

"In his opening remarks Dr. Price said: 'It has been a very great disappointment to us in making these researches to find that many of the things that we thought were so, are apparently not so. I say "apparently" because it is always within the range of possibility that there is some new phase of this problem we have not understood, we may not have had a sufficiently large number of experiments, we may not have checked our work carefully enough. However, we will leave it for you to be the jury, and I simply present to you the evidence as we have it, and you will make your own conclusions.'

"If, from the paper we don't see a better choice of medicaments and manner of using them than our present routine, we shall just have to 'carry on' as before, till we know we have something better. Dr. Price does not claim that his findings are final, and further investigation may reveal an odd missing link here and there, that will account for the good results in our experience, in spite of his findings up to date. Let us remember that within a matter of months we were informed that we were near-slackers in our dental work if we failed to convince ourselves, with X-ray proof, that our canals were all filled to the apex with guttapercha, and a well proportioned button of the same material superimposing the root end. Now, we know, that while this is clever, it is bad, inasmuch as it causes future culture pabulum at the apex for the ubiquitous 'strep.'

"Dr. Arthur Black, in his talk to our society, told us recently, that in the examination of many X-ray films, it was found that sixty-seven per cent. of teeth with root canal fillings of all kinds, showed apical infection, but that where the length of the canal filling and other characteristics suggested careful work, the percentage of trouble dropped to eight per cent. Nothing was known of the medicament, or the aseptic precautions, in this eight per cent. failure. With an approach to our best ideals in canal work, the eight per cent. may easily be reduced very close to the vanishing point.

"Dr. Price's paper should serve a valuable purpose in enabling us to make a more intelligent selection of medicaments for sterilizing root canals.

"The methods tested by Dr. Price, that gave high efficiency, all had some objectionable feature. With further investigation and experience we may retain the efficiency and overcome the objections. Formalin proved to be in the high efficiency class, but it is irritating. The Howe method with ammoniated nitrate of silver and formalin, is also in the high efficiency class, but it discolors. Oxychloride or zinc is a high efficiency root canal filling, but its hardness forbids removal. Dichloramin T. is perhaps top-notch for sterilizing infected roots, but it is also top-notch as an irritant. Yet there are those using it who have overcome its irritation. Again, there are those who employ a technic with formalin that makes its irritation negligible. Oxychloride of zinc may be so modified that it will be so friable that it can be removed from the canal if desired.

"Instead of being discouraged with our root canal work we should feel, that with Dr. Price's addition to our working knowledge of medicaments, we should be able to reach a little nearer to the goal of perfection."—*Oral Health* for April.

*Widening the Arch to Facilitate Breathing*

Nearly two years ago, a man thirty-eight years of age presented himself with the complaint that he could not breathe properly and in damp weather could not go to bed at all for fear he should suffocate. Treatment of his nose had given him only temporary relief, but had removed all obstruction which would interfere with the passage of air if the tract were wide enough.

I found his lower jaw fully developed, but his upper arch so contracted that the upper teeth closed as far inside the lowers as they should have closed outside of them.

I explained to him that while I might be able to help him a little, I doubted whether it would pay him for the trouble and expense involved, but he insisted. Using Jackson appliances, I spread his upper arch more than a half inch. Since the first three months he has had no difficulty whatever in breathing in any kind of weather, and expresses himself delighted with the results.—E. S. S., in *Dental Digest*.

